# Determinants of Digitalized Antenatal Education Use among Pregnant Women Living with Human Immunodeficiency Virus in Nigeria

**DOI:** 10.21106/IJMA_41_2025

**Published:** 2026-03-27

**Authors:** Margaret Omowaleola Akinwaare, Jonah Musa, Chizoma Millicent Ndikom

**Affiliations:** 1 Department of Maternal and Child Health Nursing, Faculty of Nursing, College of Medicine, University of Ibadan, Ibadan, Nigeria; 2 Department of Obstetrics and Gynaecology, College of Health Sciences, University of Jos, Jos Plateau, Ibadan, Nigeria

**Keywords:** Antenatal, Digital Health, Human Immunodeficiency Viruses, Maternal Health, Nigeria, Pregnancy

## Abstract

**Background and Objective:**

The introduction of digital health has been found to improve maternal health globally. However, the use and determinants of digitalized antenatal education (DAE) use among vulnerable populations are yet to be ascertained. Therefore, this study assessed the use and determinants of DAE use among pregnant women living with human immunodeficiency viruses (PWLHIV).

**Methods:**

The study adopted a descriptive design. Oyo state was randomly selected in Southwestern Nigeria, and 116 PWLHIV, who are receiving antiretroviral therapy, were recruited for the study. Of these, 93 returned completed data, which were included in the data analysis. A validated online questionnaire was used for data collection. Data analysis was done using STATA version 17. Descriptive statistics were used to assess the use, feasibility, and acceptability of DAE. Logistic regression was used to identify the determinants of DAE use.

**Results:**

Most (78 [83.9%]) of the participants reported that they have never used DAE. Similarly, 72 (77.4%) reported that they were not familiar with DAE at all. However, 84 (90.3%) of the women believed that it would be highly effective. The feasibility of DAE use is high in 83 (89%) of the participants. Furthermore, the majority of the participants, 73 (78.5%), indicated that DAE addresses their specific needs, and 77 (82.8%) of them reported being very comfortable with using DAE. The level of acceptability of DAE use is high in 73 (78.5%) of the participants. Determinants of DAE use, using adjusted odds ratio (AOR), include internet access (AOR = 1.205 with 95% confidence intervals [CI] of 0.233–6.221 and p = 0.024) and digital literacy (AOR = 14.072 with 95% CI of 2.209–89.634, and p = 0.005).

**Conclusion and Global Health Implications:**

Despite the high feasibility and acceptability of DAE in Nigeria, its use is very low among PWLHIV. Strong determinants of use include digital literacy and internet access. Thus, for PWLHIV to meet the global standard in the use of digital health, interventions to improve digital literacy and internet access in low-resource settings are recommended.

## INTRODUCTION

### Background

Antenatal education plays a key role in improving maternal and neonatal outcomes, especially for pregnant women who are infected with human immunodeficiency virus (HIV). Moreover, HIV is still a major public health issue in sub-Saharan Africa, particularly in Nigeria, where it disproportionately affects women of reproductive age.^[1]^ Antenatal education must be both accessible and sensitive to the peculiarities of this susceptible group to reduce the vertical transmission of HIV.^[2]^

Particularly in countries with limited resources, digital health innovations have shown promise as instruments to assist antenatal education by facilitating timely information transmission, treatment regimen adherence, and behavior change communication.^[3]^ Delivered through interactive voice response systems, web-based platforms, or mobile phones, digitalized prenatal education provides a scalable way to reach women who might have social, financial, or logistical obstacles to attending traditional antenatal care (ANC) programs.^[4]^ However, a number of environmental and individual factors, such as socioeconomic position, digital literacy, health beliefs, and provider engagement, affect the effective adoption and long-term usage of these technologies.^[5]^

Although mobile health (mHealth) interventions are becoming more and more popular in Nigeria, little is known about the precise factors that affect pregnant women infected with HIV in their usage of digitalized antenatal education. This disparity is crucial because these women might encounter other difficulties, such as stigma, a lack of disclosure, and disjointed care routes, all of which could influence how they use digital platforms.^[6]^ Designing more focused and inclusive digital treatments can be aided by knowledge of the determinants of use, which include demographic, psychosocial, technological, and health-system-related aspects.

In addition, age, education level, smartphone availability, technological trust, and perceived intervention utility are some of the major determinants of digital health technology use among general maternal populations that have been discovered by prior research.^[7,8]^ These results might not apply directly to HIV-positive groups, though, as they would face particular systemic and psychological obstacles. Therefore, to improve maternal and child health outcomes in this high-risk group, optimize intervention design, and increase user engagement, it is imperative to look into the determinants of digitalized antenatal education use among pregnant women living with HIV (PWLHIV) in Nigeria. The results will guide practice and policy, allowing for the creation of user-centered digital solutions to enhance maternal outcomes and lower HIV transmission from mother to child.

### Objective of the Study

The study assessed the utilization, acceptability, and feasibility of digitalized antenatal education (DAE) as well as the determinants of its use among PWLHIV.

## METHODS

This is a cross-sectional analysis of the baseline data of a large intervention study conducted to evaluate the effectiveness of DAE on the prevention of mother-to-child transmission of HIV among PWLHIV.

### Study Setting

This study was conducted across the three senatorial districts (Oyo North, Oyo Central, and Oyo South) in Oyo State, Southwestern Nigeria. Five primary healthcare centers in five different Local Government Areas of Oyo State and four state/general hospitals were selected. These healthcare facilities were selected because they provide antiretroviral therapy (ART) and counseling services for pregnant women infected with HIV.

### Study Design

This study adopted a cross-sectional analytical design to identify the determinants of DAE use among pregnant women infected with HIV. The design allows for the examination of the association between multiple independent variables and the dependent variable (use of digital antenatal education) at a single point in time.

### Study Population and Sampling

Oyo State was randomly selected by balloting among the six states in Southwestern Nigeria. Nine healthcare facilities were purposively selected because they provide ART and counseling services for pregnant women across the three senatorial districts in Oyo State. All (116) PWLHIV, who had their phone numbers documented at the selected healthcare facilities during the period of data collection, were recruited for the study. However, 93 returned completed data (giving an 80% response rate), which were included in the data analysis. The sample size was calculated according to the sample for proportions formula by Charan and Biswas^[9]^ with a 95% confidence level, 80% power, and 0.05 precision to yield a minimum representative sample of 49 participants.

### Data Collection

A validated and pretested online self-reported questionnaire developed by the researchers was adopted as a data collection tool for the study. The healthcare team (including counselors/ nurses/medical record officers/physicians/mentor mothers) was met by the researcher for modalities on data collection after being informed of the objective of the study. The participants were informed about the objective of the study by their care providers and were also informed that a group of researchers would be interacting with them on the phone.

The documented phone numbers of PWLHIV, who are receiving ART from the selected healthcare facilities, were obtained from the medical record units by the researchers. The women were contacted on the phone, the objective of the study was explained to them, and those who gave consent and had smartphones were recruited for the study. The link to the online questionnaire was shared with the participants. They were followed up with phone calls to ensure that the questionnaire was completed and also to provide guidance if needed.

### Data Analysis

Data were analyzed using STATA version 17. Descriptive analysis was used to present the feasibility, acceptability, and use of DAE, and logistic regression was done to identify facilitators/barriers of DAE use.

### Ethical Consideration

Ethical approval for the study was obtained from the Oyo State Ministry of Health Research Ethics Committee with reference number NHREC/OYOSHRIEC/10/11/22, dated June 24, 2024. Identities of research participants were kept confidential. Participants were assured that all information would be strictly for research purposes. They were also informed of their freedom to withdraw their participation at any point at no cost to them in any way. Informed consent was obtained before recruiting them for the study.

## RESULTS

### Socio-demographic Characteristics of PWLHIV in Oyo State, Nigeria

The results presented in Table 1 show the socio-demographic profile of the 93 PWLHIV. The mean age of the participants was 32.1 years, with a standard deviation of 6.0, with most, 28 (30.1%) of them between the ages of 31 and 35 years. Most 83 (89.2%) were married, more than half 50 (53.8%) were traders, and a majority 60 (64.6%) had a secondary school education.

**Table 1:** Socio-demographic characteristics of pregnant women living with HIV in Oyo State, Nigeria.

Variable	Frequency (%) (*n*=93)
Age: Mean±standard deviation	32.1±6.0
16–20	4 (4.3)
21–25	7 (7.5)
26–30	26 (28.0)
31–35	28 (30.1)
36–40	24 (25.8)
41–45	4 (4.3)
Marital status	
Married	83 (89.2)
Others	10 (10.8)
Religion	
Christianity	51 (54.8)
Islam	42 (45.2)
Ethnicity	
Yoruba	89 (95.7)
Others	4 (4.3)
Occupation	
Trading	50 (53.8)
Artisan	29 (31.2)
Civil servants	5 (5.4)
Unemployed	9 (9.6)
Highest educational attainment	
Tertiary education	21 (22.6)
Secondary education	60 (64.6)
Primary education	11 (12.8)
First pregnancy	
No	84 (90.3)
Yes	9 (9.7)
Number of previous deliveries	
None	9 (9.7)
One	23 (24.7)
Two	24 (25.8)
Three	23 (24.7)
Four	8 (8.6)
More than four	6 (6.5)

HIV: Human immunodeficiency virus

### Feasibility of DAE use among Participants

As presented in Table 2, a large proportion of the participants, 72 (77.4%), reported that they were not familiar with DAE at all. However, 84 (90.3%) of the women believed that it would be highly effective, despite the low levels of familiarity and participation. In addition, the willingness to engage with DAE is notably high, as 78 (83.9%) of the participants indicated that, although they had not participated in DAE, they would consider doing so [Table 2].

**Table 2:** Feasibility of DAE use among participants.

Variables	Frequency (%)
Level of familiarity with DAE	
Not familiar at all	72 (77.4)
Not very familiar	9 (9.7)
Somewhat familiar	8 (8.6)
Very familiar	4 (4.3)
Participation in DAE	
No, but I would consider it	78 (83.9)
Yes occasionally	6 (6.5)
Yes regularly	4 (4.3)
Accessibility to DAE	
Not accessible at all	1 (1.1)
Somewhat accessible	17 (18.3)
Very accessible	75 (80.6)
Effectiveness of DAE	
Highly effective	84 (90.3)
Not very effective	2 (2.2)
Somewhat effective	7 (7.5)
How DAE can address psychosocial needs	
Moderately well	13 (14)
Not very well	3 (3.2)
Very well	77 (82.8)

DAE: Digitalized antenatal education

**Figure 1:**
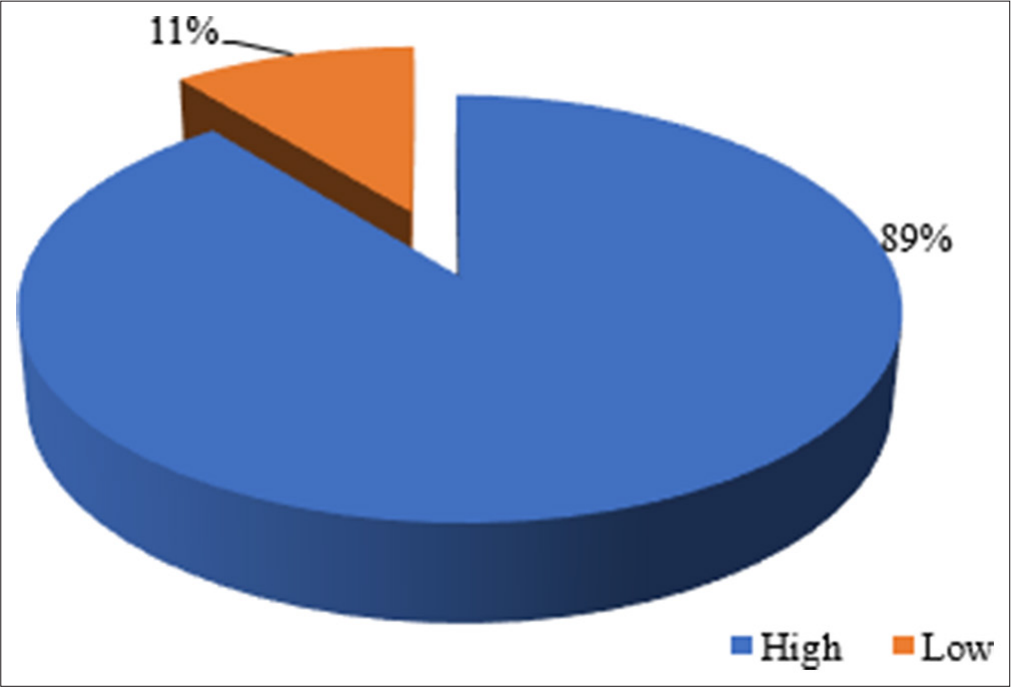
Feasibility of digitalized antenatal education use among participants.

Overall, the feasibility of DAE use is high in 83 (89%) of the participants [Figure 1].

### Acceptability of DAE use among the Participants

The results presented in Table 3 show that the majority of the participants, 73 (78.5%), indicated that DAE addresses their specific needs very well, and 77 (82.8%) of them reported being very comfortable with using DAE. Furthermore, 73 (78.5%) reported being very likely to recommend DAE when asked to indicate their willingness to recommend DAE use. In addition, the reliability of the information provided through DAE was rated as highly reliable by 82 (88.2%) of the pregnant women, and 85 (91.4%) of them rated the DAE content as being very culturally sensitive [Table 3].

**Table 3:** Acceptability of DAE use among the participants.

Acceptability	Frequency (%)
DAE addresses specific needs	
Moderately well	12 (12.9)
Not very well	8 (8.6)
Very well	73 (78.5)
Comfortable discussing sensitive topics using DAE	
Not sure	1 (1.1)
Not very comfortable	4 (4.3)
Somewhat comfortable	9 (9.7)
Very comfortable	77 (82.8)
Recommendation of DAE to others	
Not likely at all	2 (2.2)
Not very likely	8 (8.6)
Somewhat likely	10 (10.8)
Very likely	73 (78.5)
Reliability of information provided in DAE	
Highly reliable	82 (88.2)
Moderately reliable	11 (11.8)
Cultural sensitivity of DAE	
Not very culturally sensitive	1 (1.1)
Somewhat culturally sensitive	7 (7.5)
Very culturally sensitive	85 (91.4)
Continue using DAE resources	
Not likely at all	2 (2.2)
Not very likely	5 (5.4)
Somewhat likely	6 (6.5)
Very likely	80 (86)
Satisfaction with the quality of DAE	
Not satisfied at all	1 (1.1)
Not very satisfied	1 (1.1)
Somewhat satisfied	12 (12.9)
Very satisfied	79 (84.9)

DAE: Digitalized antenatal education

**Figure 2:**
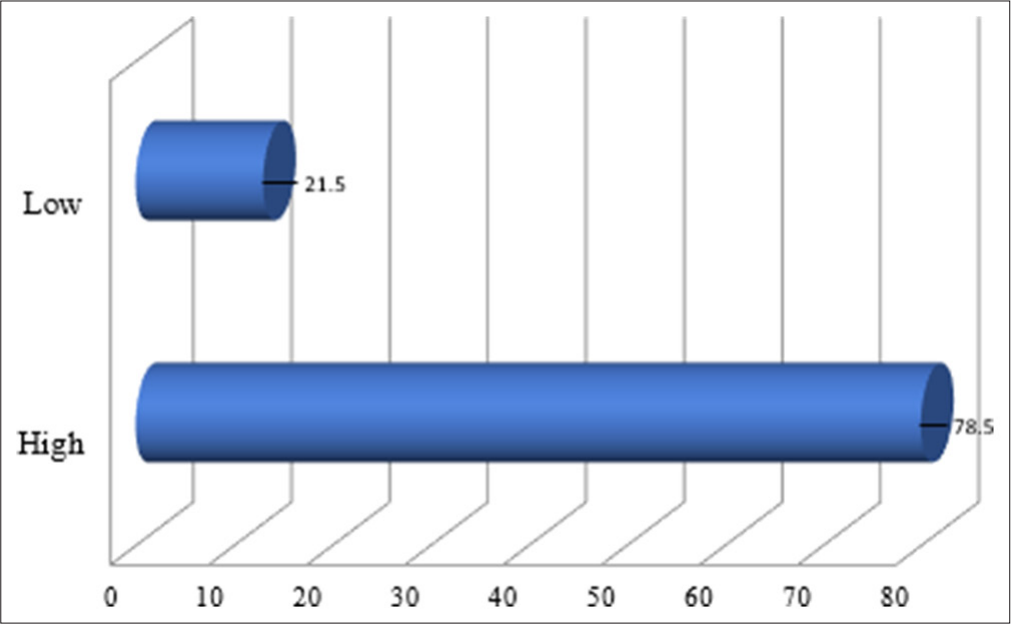
Acceptability of digitalized antenatal education among participants.

Overall, the level of acceptability of DAE use was high in 73 (78.5%) of PWLHIV [Figure 2].

### Barriers and Facilitators of DAE among Participants

The results show that one of the strongest facilitators of DAE use is healthcare provider support, with 91 (97.8%) of the women identifying it as a positive influence. Similarly, cultural sensitivity 91 (97.8%) and perceived relevance of the content 90 (96.8%) were recognized as key facilitators.

Digital literacy was also a strong facilitator 68 (73.1%). Conversely, device availability emerged as a notable barrier for 39 (41.9%) of participants. Similarly, visual and audio quality were a significant concern, with 86 (92.5%) citing it as a barrier. Social support networks represented one of the biggest barriers, with 78 (83.9%) of women reporting a lack of support as a hindrance [Table 4].

**Table 4:** Barriers and facilitators of DAE among participants.

Facilitator	Frequency (%)
Internet access	
Barrier	27 (29)
Facilitator	66 (71)
Device availability	
Barrier	39 (41.9)
Facilitator	54 (58.1)
Digital literacy	
Barrier	25 (26.9)
Facilitator	68 (73.1)
Time constraints	
Barrier	26 (28)
Facilitator	67 (72)
Healthcare provider	
Barrier	2 (2.2)
Facilitator	91 (97.8)
Partner support	
Barrier	26 (28)
Facilitator	67 (72)
Perceived relevance	
Barrier	3 (3.2)
Facilitator	90 (96.8)
Social support networks	
Barrier	78 (83.9)
Facilitator	15 (16.1)
Language preference	
Barrier	4 (4.3)
Facilitator	89 (95.7)
Cultural sensitivity	
Barrier	2 (2.2)
Facilitator	91 (97.8)
Visual and audio quality	
Barrier	86 (92.5)
Facilitator	5 (5.4)
Trustworthiness of information	
Barrier	5 (5.4)
Facilitator	86 (92.5)
Privacy concerns	
Barrier	30 (32.3)
Facilitator	63 (67.7)
Flexibility of access	
Barrier	6 (6.5)
Facilitator	87 (93.5)
Cost consideration	
Barrier	12 (12.9)
Facilitator	81 (87.1)

DAE: Digitalized antenatal education

### Crude Barriers and Facilitators of DAE Use

The analysis of the crude barriers and facilitators of DAE among PWLHIV, as presented in Table 5, reveals that digital literacy emerged as the most significant facilitator of DAE utilization. Women who were digitally literate were 15 times more likely to engage with digital antenatal education compared to those lacking digital skills (crude odd ratio (COR) = 15.529, 95% confidence intervals [CI] = 3.017–79.947, p = 0.001). Similarly, internet access was a strong and statistically significant factor (COR = 4.429, 95% CI = 1.138–17.229, p = 0.032), indicating that women with reliable internet access were 4 times more likely to use DAE than those without it. Other factors which approached statistical significance and suggest meaningful influence include women who perceived the information on DAE as trustworthy were 6.5 times more likely to use the platform (COR = 6.500, 95% CI = 0.942–44.849, p = 0.058), and those who had fewer privacy concerns were also more likely to participate (COR = 3.688, 95% CI = 0.955–14.242, p = 0.058).

**Table 5:** Crude barriers and facilitators of DAE use.

Characteristics	Crude odds ratio	95% CI	*P*‑value
Age group			
Under 20	1.000		
20–24	1.000	0.068–14.64	1.000
25–29	4.250	0.334–54.066	0.265
30–34	1.875	0.29–12.141	0.510
35–39	2.750	0.33–22.921	0.350
Occupation			
Unemployed	1.000		
Formal	0.571	0.028–11.849	0.718
Informal	1.469	0.157–13.725	0.736
Educational attainment			
Primary education	1.000		
Secondary education	0.757	0.084–6.844	0.804
Tertiary education	1.000	0.081–12.399	1.000
Ethnicity			
Yoruba	1.000		
Others	0.338	0.032–3.595	0.368
Internet access			
Facilitators	4.429	1.138–17.229	0.032
Barriers	1.000		
Device availability			
Facilitators	2.273	0.595–8.674	0.230
Barriers	1.000		
Digital literacy			
Facilitators	15.529	3.017–79.947	0.001
Barriers	1.000		
Time constraints			
Facilitators	2.952	0.777–11.216	0.112
Barriers	1.000		
Healthcare provider			
Facilitators	9.111	0.524–158.455	0.129
Barriers	1.000		
Partner support			
Facilitators	1.118	0.266–4.697	0.879
Barriers	1.000		
Perceived relevance			
Facilitators	4.500	0.37–54.673	0.238
Barriers	1.000		
Trustworthiness of information			
Facilitators	6.500	0.942–44.849	0.058
Barriers	1.000		
Privacy concerns			
Facilitators	3.688	0.955–14.242	0.058
Barriers	1.000		
Flexibility of access			
Facilitators	4.938	0.779–31.295	0.090
Barriers	1.000		
Cost consideration			
Facilitators	1.825	0.339–9.837	0.484
Barriers	1.000		

CI: Confidence intervals, DAE: Digitalized antenatal education, Significance *P*-value is 0.05

### Adjusted Barriers and Facilitators of DAE Use

Table 6 presents an analysis of two key factors, internet access and digital literacy, and their association with the utilization of DAE among PWLHIV, using odds ratios (OR), CI, and *p*-values.

**Table 6:** Adjusted barriers and facilitators of DAE.

	Odds ratio	95% CI	*P*‑value
Internet access			
Barrier	1.000		
facilitator	1.205	0.233–6.221	0.024
Digital literacy			
Barrier	1.000		
facilitator	14.072	2.209–89.634	0.005

CI: Confidence intervals, DAE: Digitalized antenatal education, Significance *P*-value is 0.05

For internet access, the OR is 1.205 with a 95% CI of 0.233 to 6.221 and P = 0.024. This indicates that women who had access to the internet were slightly more likely to use DAE than those without access. Furthermore, digital literacy shows a strong and statistically significant relationship with DAE utilization. The OR is 14.072, meaning that women who are digitally literate are over 14 times more likely to use DAE compared to those who are not. The 95% CI ranges from 2.209 to 89.634, and P = 0.005, which is well below the 0.05 threshold for significance.

## DISCUSSION

The study used a descriptive cross-sectional research design to assess the use of DAE and identified determinants of DAE use among PWLHIV.

The majority of the participants are within the reproductive age^[10]^, who affirmed that women in this age range show increased interest in digital health platforms, particularly when such tools are tailored to their time constraints and lifestyle. Most of the women were married. Olajubu et al.^[11]^ highlighted that spousal involvement positively influences the uptake of antenatal services, including digital interventions. Religious affiliation was almost evenly split between Christianity and Islam. Rahman et al.^[12]^ emphasized that religious beliefs play a key role in shaping health behaviors, and therefore, DAE content must be inclusive and respectful of diverse religious values to ensure wider acceptability. The population was largely Yoruba. The delivery of health education in local languages and with culturally familiar symbols has been shown to improve understanding and retention, as demonstrated by Musiimenta et al.^[13]^ in their study on mHealth in East Africa. With a large proportion of respondents engaged in the informal sector as traders and artisans, the DAE program must be designed for flexibility. A study by Van Pelt et al.^[14]^ noted that mHealth interventions offering asynchronous access and voice-based prompts were highly effective among informal sector workers. In terms of education, the majority of participants had completed secondary education, while some had tertiary education. According to Fan et al.,^[15]^ women with at least secondary education are more likely to engage with digital health tools effectively. The study also revealed that the majority of the respondents were not in their first pregnancy, and nearly half had two or more previous deliveries. Multiparous women, as observed by Arnold et al.,^[10]^ often seek updated information and reassurance through digital platforms, especially when previous pregnancy experiences were complicated. Their previous experiences and knowledge gained during previous antenatal care could influence their perception of DAE.

The feasibility of DAE among PWLHIV appears promising, despite significant gaps in awareness. A large proportion of participants reported being completely unfamiliar with DAE. This low level of familiarity aligns with previous research by Kebede et al.,^[16]^ who found that limited digital health awareness is a major barrier to adoption, particularly among women in lower-resource settings. Nonetheless, the same study emphasized that when digital health tools are introduced effectively, they tend to gain rapid acceptance. Remarkably, despite this unfamiliarity, a strong willingness to adopt DAE was evident. A substantial number of participants stated that while they had not yet engaged in DAE, they would consider participating in it in the future. This finding reflects the concept of “latent receptiveness” discussed by Bakibinga et al.,^[17]^ which suggests that many pregnant women, especially those living with HIV, are open to innovative educational models if these are perceived to be beneficial and non-judgmental. In terms of accessibility, the data present an encouraging scenario: most of the respondents reported that DAE is very accessible. This is consistent with findings by Osei and Boateng,^[18]^ who noted that mobile device penetration and internet availability in urban and semi-urban African communities are creating fertile ground for the use of digital maternal health tools. When evaluating perceived effectiveness, the majority of the participants believed DAE to be highly effective. Fan et al.^[15]^ highlight that perceived utility is a strong predictor of adoption, often more influential than prior use. This suggests that the benefits of DAE – such as convenience, confidentiality, and personalized content – are well recognized, even if theoretical at this stage. For PWLHIV, such features are particularly crucial, as they allow access to care without the stigma often associated with physical health facility visits.^[13]^ In addition, a striking number of participants felt that DAE would address their psychosocial needs. This highlights DAE’s potential to serve as more than just an information delivery platform. According to a study by Sinha et al.,^[19]^ digital tools that include components for mental health, peer support, and emotional reassurance are highly valued among HIV-positive mothers.

The acceptability of DAE among PWLHIV is notably high, as evidenced by multiple positive indicators. A substantial majority of participants affirmed that DAE effectively addresses their specific maternal and health-related needs. This aligns with the findings of Akintunde et al.,^[20]^ who emphasized that tailored digital health content, especially when customized to reflect the lived experiences of vulnerable groups like women living with HIV, can significantly improve perceived relevance and user satisfaction. When it comes to discussing sensitive topics such as HIV status, stigma, sexual health, or mental well-being, most of the women indicated that they felt very comfortable using DAE platforms. According to Musiimenta et al.,^[13]^ digital platforms provide a safe environment for women to explore personal health issues without fear of judgment or disclosure, thereby fostering greater openness and engagement. In terms of advocacy, the willingness to recommend DAE to others was high, with most stating they were very likely to do so. A similar pattern was found in a study by Bakibinga et al.,^[17]^ which showed that recommendation plays a key role in scaling digital health solutions among African women, especially when peer approval reinforces trust and credibility. The reliability of information provided through DAE was endorsed by the majority of the respondents, who rated it as highly reliable. Sinha et al.,^[19]^ argue that the trustworthiness of content, backed by validation from health professionals, is a strong determinant of the acceptability of digital interventions. For PWLHIV, accurate and reliable content also reduces confusion and misinformation, which are common barriers to health-seeking behaviors. Cultural relevance is another area where DAE appears to perform exceptionally well. A striking number of participants described the content as very culturally sensitive. This finding supports the conclusions of Osei and Boateng^[18]^ who asserted that culturally tailored interventions significantly enhance user engagement and acceptability, especially in settings where traditional beliefs heavily influence maternal behaviors. Regarding continued usage, most of the participants reported that they were very likely to keep using DAE resources. According to Fan et al.,^[15]^ digital health platforms that are intuitive, relevant, and adaptable are more likely to retain users long-term. For women managing both pregnancy and HIV, ongoing access to support tools can provide stability and confidence throughout the perinatal period. Finally, satisfaction with the overall quality of DAE was impressively high, with most of the women reporting being very satisfied. Kebede et al.^[16]^ emphasize that satisfaction plays a pivotal role in digital health success, as it directly influences future participation, adherence to advice, and the likelihood of recommending the service to others.

The adoption of DAE among PWLHIV is shaped by a complex interplay of facilitators and barriers. One of the strongest facilitators identified was support from healthcare providers, with almost all of the respondents acknowledging this as a key motivator. This finding is consistent with Nguyen et al.,^[21]^ who emphasized that endorsement by healthcare professionals enhances the credibility and perceived safety of digital health tools, particularly for vulnerable populations. Similarly, perceived relevance and cultural sensitivity emerged as important facilitators. According to Osei and Boateng,^[18]^ interventions that align with users’ lived experiences and cultural beliefs are more likely to be accepted and sustained. Digital literacy was identified as a significant facilitator, with most of the respondents indicating they had the skills to interact with digital platforms. This finding corresponds with Musiimenta et al.,^[13]^ who noted that digital competence is a foundational requirement for the successful adoption of mHealth interventions. Other important facilitators include internet access and time availability. Fan et al.^[15]^ argued that access to reliable internet and autonomy over 1’s time are essential for the feasibility of any digital health initiative.

Despite these enabling factors, several barriers persist. Device availability was cited as a constraint by almost half of the respondents. This underscores a significant infrastructural gap, confirmed by Amoakoh-Coleman et al.^[22]^ who reported that device unavailability is one of the biggest obstacles to mHealth adoption in sub-Saharan Africa. This calls for effective offiine interventions for childbearing women. Thus, offiine interventions and a community-based device sharing model are highly recommended for the implementation of DAE among pregnant women. Another major obstacle was the quality of audio and visual content, which almost all of the participants identified as a challenge. This finding aligns with Gurman et al.,^[23]^ who stressed that poorly designed user interfaces and low-quality content reduce user satisfaction and trust in digital interventions. Lack of social support networks was also a significant barrier, with the majority of the participants noting it as a limitation. Social isolation is a known issue for women living with HIV, and Sinha et al.^[19]^ argue that the absence of peer or family support can negatively affect maternal healthcare utilization. Privacy concerns were reported by almost one-third of the participants, highlighting a critical issue for women managing a stigmatized condition like HIV. According to Kebede et al.,^[16]^ fear of unintended disclosure is a common deterrent to using digital health services among HIV-positive populations. Although cost considerations were a barrier for only a few of the respondents, affordability remains a relevant factor, especially in low-income households. This supports findings by Bakibinga et al.,^[17]^ who found that digital health tools offer long-term savings on transportation and clinic visits. Partner support was reported as a facilitator by most of the participants, but also presented a barrier for a few of them. This highlights the gendered dynamics influencing digital health adoption. Akinfaderin-Agarau et al.^[6]^ noted that male partner approval can significantly shape women’s participation in health programs. Additional facilitators identified in this study include the trustworthiness of information, language preference, and flexibility of access. These findings mirror Schnall et al.^[24]^ who emphasized that trust, linguistic appropriateness, and on-demand availability are fundamental features that enhance the utility and credibility of digital maternal health tools.

Conclusively, PWLHIV have positive dispositions to DAE use as displayed by their perceived effectiveness of DAE, even though many of them do not have access to it, and are not familiar with it. They are optimistic about using DAE. Therefore, policymakers and stakeholders in maternal health and HIV prevention should promote facilitators of DAE use among pregnant women and plan interventions that could mitigate the identified barriers to improve digital health in low-resource settings like Nigeria. Furthermore, program implementers might need to consider offiine interventions to be able to reach women who do not have access to internet-enabling devices, and those who have restricted or no internet access. In addition, future research could investigate how digital literacy could be improved among pregnant women, as well as the effectiveness of offiine interventions that could promote PMTCT.

## CONCLUSION AND GLOBAL IMPLICATIONS

The use of DAE is feasible and acceptable among PWLHIV in Nigeria. However, effective interventions such as training programs for digital literacy (especially for childbearing women), subsidies for smartphones, and integrating DAE into routine HIV and maternal health care to promote its facilitators are highly recommended. In addition, the content of such interventions should be culturally sensitive to reduce stigmatization among vulnerable populations, such as PWLHIV. A lot of efforts still need to be put in place if PWLHIV in low-resource settings like Nigeria will meet the global standards in the utilization of digital health.

### Limitations of the Study

The design of the online data collection tool did not account for the completeness of the instrument before submission.

Hence, about one-fifth of the participants had incomplete data, which could not be included in the data analysis. Furthermore, the wide CI observed could be due to over-powering of the study, given the large sample size relative to the originally calculated minimum. In addition, the study only included women with smartphones and documented phone numbers, which may have introduced some selection bias. However, since the study’s objective is to evaluate the use of smartphones for accessing antenatal education, only women with smartphones were included in the study. Furthermore, the researchers intentionally avoided physical contact with the participants to minimize stigmatization; this is the reason for using the documented phone numbers at the health facilities.

### Key Messages

(1) The use of DAE is highly feasible and acceptable to PWLHIV in Nigeria. Hence, the availability and accessibility to such education will reduce stigmatization associated with in-person education, and thereby promote prevention of mother-to-child transmission of HIV. (2) Effective interventions to improve digital literacy among PWLHIV will increase digital health utilization.

## Acknowledgments

The authors acknowledge the support of Dr. Claudia Hawkins of Robert J Harvey MD Institute of Global Health, Northwestern University, Chicago, IL, USA, for providing an enabling environment during the manuscript writing facilitation for fellows. The research assistants and the ad hoc research assistants are sincerely appreciated for their commitment during the data collection. Special thanks to the APIN Public Health Initiatives in Oyo State, Nigeria, and all the Local Action Committee on AIDS (LACA) officers in Oyo State for their cooperation during the data collection.

## COMPLIANCE WITH ETHICAL STANDARDS

### Conflicts of interest

The authors declare no competing interests.

### Financial Disclosure

Not applicable.

### Ethics Approval

The study received ethical approval from the Oyo State Ministry of Health Research Ethics Committee with approval number NHREC/OYO/SHRIEC/10/11/22, dated June 24, 2024. Informed consent was obtained from the participants.

### Use of Artificial Intelligence (AI)-Assisted Technology for Manuscript Preparation

The authors confirm that there was no use of artificial intelligence (AI)- assisted technology for assisting in the writing or editing of the manuscript and no images were manipulated using AI.

### Disclaimer

None.
